# Epigenetic regulation of epithelial-mesenchymal transition: focusing on hypoxia and TGF-β signaling

**DOI:** 10.1186/s12929-020-00632-3

**Published:** 2020-03-02

**Authors:** Yueh-Te Lin, Kou-Juey Wu

**Affiliations:** 1 Cancer Genome Research Center, Chang Gung Memorial Hospital at Linkou, Gueishan Dist., Taoyuan, 333 Taiwan; 2 Institute of Cellular and Organismic Biology, Academia Sinica, Taipei, 115 Taiwan; 3 Inst. of Clinical Medical Sciences, Chang Gung University, Taoyuan, 333 Taiwan

**Keywords:** Epithelial-mesenchymal transition, Hypoxia, TGF-β, Epigenetic regulation, HIF-1α, HDAC3, WDR5, H3K4Ac, ChIP, LSD1, H2A.Z

## Abstract

Epithelial-mesenchymal transition (EMT) is an important process triggered during cancer metastasis. Regulation of EMT is mostly initiated by outside signalling, including TGF-β, growth factors, Notch ligand, Wnt, and hypoxia. Many signalling pathways have been delineated to explain the molecular mechanisms of EMT. In this review, we will focus on the epigenetic regulation of two critical EMT signalling pathways: hypoxia and TGF-β. For hypoxia, hypoxia-induced EMT is mediated by the interplay between chromatin modifiers histone deacetylase 3 (HDAC3) and WDR5 coupled with the presence of histone 3 lysine 4 acetylation (H3K4Ac) mark that labels the promoter regions of various traditional EMT marker genes (e.g. *CDH1*, *VIM*). Recently identified new hypoxia-induced EMT markers belong to transcription factors (e.g. SMO, GLI1) that mediate EMT themselves. For TGF-β-induced ΕΜΤ, global chromatin changes, removal of a histone variant (H2A.Z), and new chromatin modifiers (e.g. UTX, Rad21, PRMT5, RbBP5, etc) are identified to be crucial for the regulation of both EMT transcription factors (EMT-TFs) and EMT markers (EMT-Ms). The epigenetic mechanisms utilized in these two pathways may serve as good model systems for other signalling pathways and also provide new potential therapeutic targets.

## Introduction

Epithelial-mesenchymal transition (EMT) is one of the initial and crucial mechanisms causing cancer metastasis [1–3]. EMT is originally a developmental program that has been utilized by tumor cells to facilitate their migration, invasion, and final colonization in distant sites, resulting in distant metastasis that causes poor prognosis of cancer patients [1–3]. The simple developmental transition would include the metamorphosis of epithelial cells into mesenchymal cells [1–3]. During the process, the coupling of repression of epithelial markers and activation of mesenchymal markers is constantly observed [1–3]. However, this transition process is reversible since tumor cells going through EMT will adopt the reversible proces of mesenchymal-epithelial transition (MET) when the tumor cells migrate into distant sites so they can remain epithelial in origin and exert more malignat behaviros [4].

The major signaling pathways that can trigger the process of EMT include hypoxia, TGF-β, Notch, Wnt, integrins, etc [1–3]. While these outside signalings trigger the activities of intracellular signaling molecules (e.g. Smad, HIF-1α, MAPK, NF-κB, β-catenin, NICD, etc), these signaling molecules can subsequently activate different EMT transcription factors (EMT-TFs) to induce the EMT program, including repression of epithelial markers and activation of mesenchymal markers, which are designated as EMT markers (EMT-Ms) [1–3]. In addition, induction of cancer stemness and regulation of immune suppression are also observed during the EMT process [1–3, 5–9]. Therefore, with all these mechanisms causing tumor progression, the process of EMT confer tumors with malignant property, drug resistance, cancer stemness, ability to supress immune surveillance, etc [1–3, 5–9]. All these phenotypes induced by EMT will obviously facilitate the agressiveness of tumor cells and explain the treatment resistance of different tumor types in their terminal stage [1–3, 5–9]. Finally, the non-redundant functions of EMT-TFs have been recently emphasized [10]. Due to the differential expression patterns and levels in different tumor types, different EMT-TFs would have different functions as well as different target genes in a context-dependent manner [10]. Therefore, this aspect needs to be considered in reaching the conclusions of the functions of different EMT-TFs [10].

## Regulation of EMT transcription factors (EMT-TFs) vs. EMT markers (EMT-Ms)

The regulation of EMT can be divided into regulation of EMT transcription factors (EMT-TFs) such as Snail, Twist1, Slug, SIP1, ZEB1, etc and regulation of EMT markers (EMT-Ms) such as epithelial and mesenchymal markers [1–3]. In addition, immune regulation and cancer stemness properties could be listed under the category of EMT markers [1, 5–9]. Most of the known literature address the regulation of EMT-Ms, but less on the regulation of EMT-TFs [1–3, 10]. Other than the signaling molecules regulating EMT, epigenetic regulation is an important aspect of regulation [11, 12]. Different chromatin modifiers, non-coding RNAs, RNA splicing events, and DNA methylation/demethylation are major players in the epigenetic regulation of EMT [11–13]. For the review articles commenting on EMT, some of the epigenetic mechanisms have been presented in previous literature [11, 12]. Therefore, this review will focus more on the recent progress of epigenetic mechanisms. In addition, we will only focus on two major pathways that regulate EMT: hypoxia and TGF-β-induced signaling. We will also summarize the most recent results on chromatin modifiers but not touch on non-coding RNAs or RNA splicing events to simplify the issue. We believe that the recent results from these two pathways will be good model systems to study the epigenetic regulation of EMT mediated by other signaling pathways.

For epigenetic regulation of EMT, polycomb repressor complexes (PRCs), histone deacetylases (HDACs), and histone lysine demethylases (KDMs) are notable examples [11, 12]. DNA demethylation by TET1 has been reported to be involved in hypoxia-induced EMT [13]. Previous review articles already described the contribution of different epigenetic factors to the regulation of EMT [11, 12, 14, 15], including transcription co-regulators, DNA methyltransferases (DNMTs), Histone acetyltransferases/histone deacetylases (HATs/HDACs), histone methyltransferases/lysine demethylases (HMTs/KDMs), and other chromatin modifiers. Detailed descriptions of the relevant mechanisms can be found in these review articles [11, 12, 14, 15].

## Epigenetic regulation of hypoxia-induced EMT: focusing on H3K4Ac histone mark and immune suppression

Hypoxia is one of the crucial microenvironmental factors that induces cancer metastasis [16, 17]. Hypoxia-inducible factor-1α (HIF-1α) stabilization under hypoxic condition regulates many critical steps of metastasis, and one of the critical steps regulated by hypoxia is EMT [16–18]. In addition to regulating EMT markers, hypoxia/HIF-1α is one of the major events regulating different EMT transcriptional regulators, including Snail, Twist1, ZEB1, ZEB2, SIP1, *etc* [16–18]*.*

For regulation of EMT markers (EMT-Ms) during hypoxia, it has been shown that the interplay between chromatin modifiers histone deacetylase 3 (HDAC3) and WDR5 coordinately regulates the repression of epithelial genes and activation of mesenchymal genes [19]. From this study, the histone mark histone 3 lysine 4 acetylation (H3K4Ac) has been shown to label the promoters of certain EMT marker genes including E-cadherin (*CDH1)*, vimentin (*VIM),* etc [19]. The presence of both H3K4me3 (an activation histone mark) and H3K27me3 (a repression histone mark) is observed in the promoters of EMT marker genes (EMT-Ms) during hypoxia-induced EMT [19]. This bivalent presence of activation and repression histone marks represent a status of “poised transcription” that allows EMT markers to go from active to repressed status and come back to active status in a more flexible manner. Recent results using chromatin immunoprecipitation (ChIP) by anti-H3K4Ac antibodies followed by whole genome sequencing analysis identified new putative EMT marker genes that respond to hypoxia and are regulated by HDAC3 [20]. These new EMT markers labeled by H3K4Ac are shown to be involved in *in vitro* migration and invasion [20]. Among these new EMT markers identified, glioma-associated oncogene homolog 1 (GLI1) and smoothened SMO) that belong to the Hedghog pathway are shown to be regulated by HDAC3. Together they contribute to *in vitro* migration/invasion, and serve as a marker of prognosis in head and neck cancer patients [20]. It is interesting that GLI and SMO as transcription regulators are labeled by H3K4Ac and can be categorized as EMT markers (EMT-Ms) [19, 20]. Other factors including FOXF1 and Bmi1 are also transcription regulators that promote tumor progression and tumor stemness [6, 20]. FOXF1 can activate Snail to promote EMT in colorectal cancer cells [21]. This result highlight the significance of H3K4Ac histone mark that can be a marker for EMT marker genes or EMT transcription factors, which blurred the categorization between EMT-Ms and EMT-TFs since Bmi1 and FOXF1 may be listed under the category of EMT-TFs. In addition, H3K4Ac labeling of hedgehog signaling molecules is consistent with previous findings that hypoxia induces sonic hedgehog signaling [22]. Another interesting finding out of this result is that the binding motifs identified through H3K4Ac ChIP-seq also contains Ikaros binding site, indicating that Ikaros may play an accessory role in labeling H3K4Ac [20]. After removal of H3K4Ac by HDAC3, Ikaros may cease to play a repressor role to allow these EMT-Ms to go through EMT [20].

For H3K4Ac histone mark that labels the EMT marker genes, a relevant result from budding yeast studies indicates its role in flexible gene regulation [23]. The presence of H3K4Ac mark (mediated by *Mst1* in budding yeast) reduced *Chp1*/*Clr4* affinity to H3K9me2 and switched to *Chp2*/*Swi6* binding to H3K9me2 and this event presents a chromodomain switch to allow heterochromatin reassembly [23]. Therefore, the presence of H3K4Ac in budding yeast allows for regulation of genes alternating between active and inactive states [23]. In mammalian cells, H3K4Ac removal by HDAC3 in epithelial gene promoters will be substituted by deposition of H3K4me2/3 (an active histone mark) [19]. In these promoters, the simultaneous presence of H3K27me3 (a repressive histone mark) presents a bivalent status of “poised transcription” to allow for later MET and facilitate the smooth progress of EMT-MET in tumor cells [19]. Another “poised transcription” example is the presence of bivalent chromatin configuration (H3K4me3 and H3K27me3) in the ZEB1 promoter at non-cancer stem cell (CSC) status [24]**.** The significance of this bivalent chromatin status is that it is adopted by tumor cells at non-CSC status that can be converted to CSC status in response to microenvironmental signals [24]. Therefore, bivalent chromatin configuration can occur in both EMT-Ms and EMT-TFs [19, 20, 24]. In addition, H3K4Ac mediated by *Gcn5* and *Rtt109* in budding yeast is enriched at promoters of actively transcribed genes and located upstream of H3K4me3, and this pattern is conserved in human cells [25]. Along the same scenario, H3K4Ac is associated with deregulated cancer related pathways, especially with estrogen receptor (ER) signaling and EMT pathway in breast cancer cells [26]. H3K4Ac is a better predictor of genes undergoing active and poised transcription than H3K4me3 mark and can be used to predict early stage of tumor progression [26]. As demonstrated in budding yeast that *Gcn5* and *Rtt109* mediate H3K4Ac, TIP60 is shown to be the H3K4 acetyltransferase in mammalian cells [27]. Low expression of TIP60 promotes breast cancer progression in ER-negative tumors [27]**,** consistent with removal of H3K4Ac during hypoxia-induced EMT that predates tumor progression [12, 19]. Along the same line, loss of H3K4Ac has been shown to correlate with melanocytic malignant transformation [28].

Hypoxia has been demonstrated to mediate immune suppression so tumors cells under hypoxia can gain the advantage of escaping immune surveillance [7, 8]. HIF-1α has been shown to activate different molecules to achieve this goal, including activation of anti-apoptosis genes in tumor cells, activation of PD-L1 to escape CTL-mediated killing, activation of CD39/CD73 to accumulate adenosine and inhibit CTL, induction of autophagy to inhibit NK cell mediated lysis, activation of CD47 to block phagocytosis by macrophage, and induction of chemokines/cytokines to recruit T regulatory cells (Tregs) and marrow derived stem cells (MDSCs) to inhibit tumor immunity [7, 8]. Recent results showed that hypoxia activates miR25/93 to repress the cGAS-STING pathway and mediate immunesuppression [9]. Among these mechanisms to evade immune surveillance, activation of PD-L1 and CD47 has been linked to EMT-TFs and DNA methylation. In addition to the activation by HIF-1α, activation of PD-L1 and CD47 can be mediated by ZEB1 and Snail/ZEB1, respectively [29, 30]. Furthermore, overexpression of PD-L1 during EMT requires the decreased DNMT1 levels to cause PD-L1 promoter demethylation in lung cancer cells [31]. In addition to the multiple mechanisms that regulate immune surveillance by hypoxia and EMT-TFs, we believe that more epigenetic mechanisms will be discovered in the future to further delineate the observations of tumor-mediated immune regulation.

## Epigenetic regulation of TGF-β-induced EMT: focusing on regulation of EMT-Ms vs. EMT-TFs

TGF-β signaling is important developmentally for morphogenesis, cell proliferation, differentiation, epithelial-mesenchymal transition, regeneration, and immune regulation [32, 33]. For disease implications, TGF-β signaling plays a crucial role in tumor metastasis and progression (through promotion of EMT), tumor immunity, and organ fibrosis [32, 33]. Epigenetic reprogramming through various mechanisms during TGF-β-induced signaling has been summarized [11, 12, 32]. Changes of different histone marks (H3K27me3, H3K9me3, etc) and DNA methylation have been observed during the process of TGF-β-induced EMT [34]. For the regulation of different EMT-TFs and EMT-Ms, a variety of mechanisms have been demonstrated [34] and there is no doubt that new mechanisms of epigenetic regulation will be identified using new approaches or technologies.

Smad proteins have been shown to recruit chromatin and DNA modifiers to regulate gene expression (for detailed mechanisms, please see [32, 33]). Another function of Smad proteins is that Smad proteins associate with histone reader to silence chromatin but keep the chromatin status “poised’ in ES and progenitor cells [32, 33]. In this example, TRIM33 binds to Smad2/3 to form a chromatin reader that reads a repressive but poised histone mark, H3K9me3 [32, 33]. The binding of TRIM33-Smad2/3 complex to H3K9me3 will displace heterochromatin protein 1 (HP1) and allow subsequent activation of mesendoderm differentiation genes such as GSC and MIXL1 by the Smad4-Smad2/3 complex triggered by Nodal signaling [32, 33]. Another regulation of Smad2/3 proteins showed that profilin-2 interacts with HDAC1 to inhibit the binding of HDAC1 to the promoters of Smad2 and Smad3, leading to their activation and enhancing the TGF-β-induced EMT and angiogenesis in lung cancer cells [35]. In addition, BRD7 interacts with Smad3/4 through its N-terminal Smad-binding domain to enhance TGF-β-induced signaling and EMT phenotypes [35]. BRD7 simultaneously binds to acetylated histones to promote Smad-chromatin association and associates with p300 to increase Smad transcriptional activity and promote TGF-β transcriptional activity and EMT [36].

Global epigenetic reprogramming during TGF-β-induced EMT shows the decrease in H3K9me2 and increase in H3K4me3/H3K36me3 [37]. These changes are dependent on LSD1 and loss of LSD1 mitigates the phenotypes of TGF-β-induced EMT, including cell migration and chemoresistance [37]. In addition, these chromatin changes are specific to large organized heterochromatin K9 modifications (LOCKs), supporting the notion that TGF-β-induced EMT is caused by reprogramming of specific chromatin domains [37]. However, other chromatin modifiers are required to regulate the expression of E-cadherin and vimentin since LSD1 alone cannot regulate this process [37]. These results indicate that histone H3K4me3/H3K36me3 changes in response to TGF-β are targeted to specific genes (e.g. motility genes) and that the functional outcome of these histone mark changes is highly context-dependent [37].

Similar to the labeling of EMT-Ms with H3K4Ac in hypoxia-induced EMT, the histone variant H2A.Z has been discovered to label the EMT-Ms in TGF-β-induced EMT and becomes a master regulator [38]. TGF-β induces the repression of H2A.Z and knockdown of H2A.Z mimicks the TGF-β-induced EMT [38]. Mechanistically, H2A.Z is located in the -1 nucleosome position upstream of TSS in the epithelial genes and removal of H2A.Z represses epithelial gene expression. In contrast, H2A.Z is located in the +2 nucleosome position downstream of TSS in the mesenchymal genes and removal of H2A.Z upregulats mesenchymal gene expression [38]. This result supports the initial observation that EMT-Ms in hypoxia-induced EMT are labeled with a histone mark (H3K4Ac) and removal of this mark is required for hypoxia-induced EMT [19]. The role of H2A.Z in TGF-β-induced EMT is similar to H3K4Ac in the labeling of EMT-Ms. It will be interesting to search for the enzyme that removes H2A.Z under TGF-β stimulation, similar to the role of HDAC3 in the removal of H3K4Ac during hypoxia-induced EMT [19].

It is interesting that a subunit of the cohesion complex, Rad21, plays a role in the induction of TGF-β1 [39]. Rad21 is expressed in epithelial breast cancer cells and knockdown of Rad21 promotes EMT through releasing three dimensional chromatin loop structure and opening the chromatin configuration of TGF-β1 and ITGA5 promoters as well as other mesenchymal genes that correlate with the gene expression pattern of stem cell-like cancer cells [39]. This result points to the important role of cohesion-mediated chromatin architecture in the regulation of EMT-cell fate determination [39].

Other molecules that enhance TGF-β signaling are Protein arginine methyltransferase 5 (PRMT5) and MEP50 [40]. PRMT5-MEP50 complex catalyzes histone mono- and dimethylation of chromatin at key EMT genes to potentiate TGF-β-induced response in lung cancer and breast cancer cells [40]. Mechanistically, PRMT5-MEP50 complex mediates H3R2 methylation to activate EMT activator genes (vimentin, Snail, Slug) through recruitment of WDR5 and cause H3K4 methylation; where this complex also mediates H4R3 methylation to repress metastasis suppressor genes (e.g. E-cadherin, GAS1) [40], providing an example of histone arginine methylations to control gene expression. For high mobility group proteins (HMGs), HMGA20 is shown to be a subunit of the LSD1-CoREST complex for Snail to repress epithelial gene expression during TGF-β-induced EMT [41]. The other HMG protein, HMGA2, together with Smads co-regulates Snail expression [42]. Furthermore, overexpression of HMGA2 causes repression of E-cadherin through recruiting the binding of DNMT3A and methylation of the E-cadherin promoter [43]. Treatment with Azacytidine re-activates the expression of E-cadherin [43]. This result demonstrates the sustained repression of E-cadherin by HMGA2 through promoter methylation [43], and provides a good example of a molecule regulating both EMT-TFs and EMT-Ms. Other results show that Slug is induced by TGF-β to mediate EMT in hepatoma cells, in which G9a and HDACs are crucial for Slug-induced E-cadherin repression [44]. Another result shows that EED, a component of the PRC2 complex to mediate H3K27 trimethylation, is induced by TGF-β to mediate EMT through repression of E-cadherin and miR-200 family members, leading to activation of ZEB1 and ZEB2 [45]. The mechanism is mediated through H3K27 methylation of the promoters of E-cadherin and miR-200 family members by EZH2/EED [45]. A similar mechanism is shown in the case of JARID2, a component of the PRC2 complex [46]. JARID2 is also induced by TGF-β to repress E-cadherin expression through occupancy of JARID2 in the promoters of E-cadherin and miR-200 family members, which controls PRC recruitment and histone methylation in lung cancer and colon cancer cells [47]. In prostate cancer cells, TGF-β induces Snail expression through increasing H3K4me3 enrichment and RbBP5 binding in the promoter of Snail through further recruitment of Smad2/3 and CBP around the transcription start site of Snail [47]. TGF-β also induces KDM6B to remove H3K27me3 mark on the Snail promoter and activate Snail expression in breast cancer cells [48]. A similar result showed that TGF-β decreases the levels of EZH2 and H3K27me3 globally in SKOV3 ovarian cancer cells [49]. In addition, activation of ZEB2 expression by TGF-β is mediated through the decreased EZH2 and H3K27me3 levels in the promoter of ZEB2 [49]. In contrast, through multi-omics approaches by comparing proteome, phosphoproteome, and histone modifications during TGF-β-induced EMT, Erk signaling and H3K27me3 mark are shown to be upregulated during TGF-β-induced signaling [50]. Therefore, inhibitors of Erk activity and EZH2 may be utilized to inhibit TGF-β-induced malignant phenotypes [50]. Another important player in TGF-β-induced EMT is SETDB1, a H3K9 histone methyltransferase. SETDB1 has been implicated in promoting tumorigenesis in melanoma and lung cancer [51, 52]. However, SETDB1 plays a different role in the non-invasive tumor mass vs. invasive front during TGF-β-induced signaling [53]. In the non-invasive tumor mass, TGF-β induces the association of SETDB1 with Smad3 to repress the expression of Snail through mediating H3K9 methylation in the promoter of Snail [53]. In contrast, in the invasive front of tumor cells undergoing TGF-β-induced EMT, SETDB1 expression is repressed by TGF-β to de-repress the Smad3-SETDB1-mediated repression of the Snail promoter [53]. Therefore, SETDB1 expression represents the balance between the chromatin modification status (H3K9 acetylation vs. H3K9 methylation) in the Snail promoter in different cellular contexts [53]. A similar mechanism (association between Smad3 and SETDB1 to repress gene expression) has also been shown to suppress lung cancer metastasis through repressing ANXA2 expression and repress IL-2 gene expression in activated T cells [54, 55]. The examples of SETDB1 in the context of TGF-β signaling represent the pleiotropic effects of TGF-β during cancer metastasis and immune regulation [53–55].

One notable example of epigenetic regulation of EMT transcription regulators (EMT-TFs) is the discovery of UTX, the H3K27 demethylase, that regulates EMT [56]. Knockdown of UTX induces EMT through recruiting c-Myc/p300 to the promoters of EMT-TFs in breast cancer cells. UTX cooperates with LSD1 (and HDAC1 and DNMT1) to compete with the MLL histone methyltransferase complex and disrupt the recruitment of c-Myc and p300, resulting in the inhibition of H3K4 methylation in the promoters of EMT regulators such as Snail, ZEB1, and ZEB2 [56]. The promoter of CDH1 is also repressed through the same mechanism. Therefore, UTX serves as a tumor suppressor to negatively regulate EMT-induced CSC-like properties by epigenetically repressing EMT-TFs in breast cancer cells [56].

## Intermediate states of EMT and its epigenetic regulation

Recent progress in EMT research has shown that there exist intermediate states of EMT [1, 2, 57, 58]. The intermediate states can be divided into “early hybrid EMT state”, “hybrid EMT state”, and “late hybrid EMT state” [57, 58]. It has been shown that tumor cells possessing the highest metastatic potential are the tumor cells positioned in the intermediate EMT states [1, 2, 57]. A specific set of transcription factors have been identified in these “hybrid EMT states” that are responsible for transcriptional and epigenetic landscapes of these tumor cells going through EMT transition states (for detailed descriptions, please see ref. [57, 58]. However, the detailed epigenetic changes during these intermediate states of EMT have not been well characterized [57]. In contrast, dynamic and reversible changes of DNA methylation have been observed during the EMT process and have been associated with transcription regulation of EMT related genes [59]. It will be reasonable that the epigenetic changes and DNA methylation changes observed during the intermediate states of EMT can interact and cross-talk with each other to ensure the smooth transition of tumor cells going through these transition states of EMT. Further efforts will be required to delineate the detailed molecular mechanisms and the integration of transcriptional and epigenetic regulations.

## New approaches, future directions and therapeutic implications

Other than the traditional molecular and cell biological analysis, a recent approach was performed using single cell RNA-sequencing to profile MCF10A cells going through a spontaneous spatially determined EMT in the presence or absence of TGF-β [60]. Trajectory analysis showed that continuous waves of gene expression could be observed, indicating the continuum nature of EMT in contrast to the conventional “partial” stages of EMT [60]. From this analysis, KRAS emerges as the critical molecule to control the exit of epithelial stage to turn into mesenchymal phenotype [60]. Using a pooled single-cell CRISPR-Cas9 screening, EMT-associated receptors and transcription factors, including regulators of KRAS, were identified whose loss inhibited the progress of EMT [60]. Further experiments showed that KRAS effector MEK and its upstream regulators EGFR and MET represent regulatory “checkpoints” in the EMT continuum [60]. This result represents a state of the art approach using single-cell RNA-sequencing analysis and single-cell CRISPR-Cas9 screening to obtain a full and continuous spectrum examination of the EMT process [60].

In addition to single-cell RNA sequencing analysis, chromatin changes can be analyzed by different new technologies, including assay for transposase-accessible chromatin followed by sequencing (ATAC-seq), chromosome conformation capture (3C), Hi-C, etc [61–63]. Therefore, the EMT process can be further analyzed in more details and through different angles. These new approaches will certainly shed more light on the EMT process mechanistically and will provide novel concepts. Finally, a recent epitranscriptomics approach through profiling RNA *N*^6^-methyladenosine methylation (m6A) showed that m6A plays a role in EMT by regulating the translation of Snail [64]. RNA m6A modification should be a new direction that warrants attention in the field of EMT.

The chromatin modifiers identified during the EMT process induced by either hypoxia or TGF-β will present good targets for future therapeutic purposes. For HDAC3, well established HDAC inhibitors have been used clinically [15, 19]. EZH2 inhibitor has also been used in clinical trials [15]. Other targets such as profilin-2, EED, RbBP5, and JARID2 can all be good epigenetic targets for new drug development [35, 45–47]. Erk inhibitor can be combined with EZH2 inhibitor to inhibit TGF-β-induced EMT [50]. In the case of HMGA2, recruitment of DNMT3A would warrant the usage of 5-Azacytidine to inhibit HMGA2-induced EMT [43]. It is obvious that development of new epigenetic drugs will be beneficial for patients with different tumor types that may respond to different kinds of epigenetic therapy.

## Conclusion

**Fig. 1:**
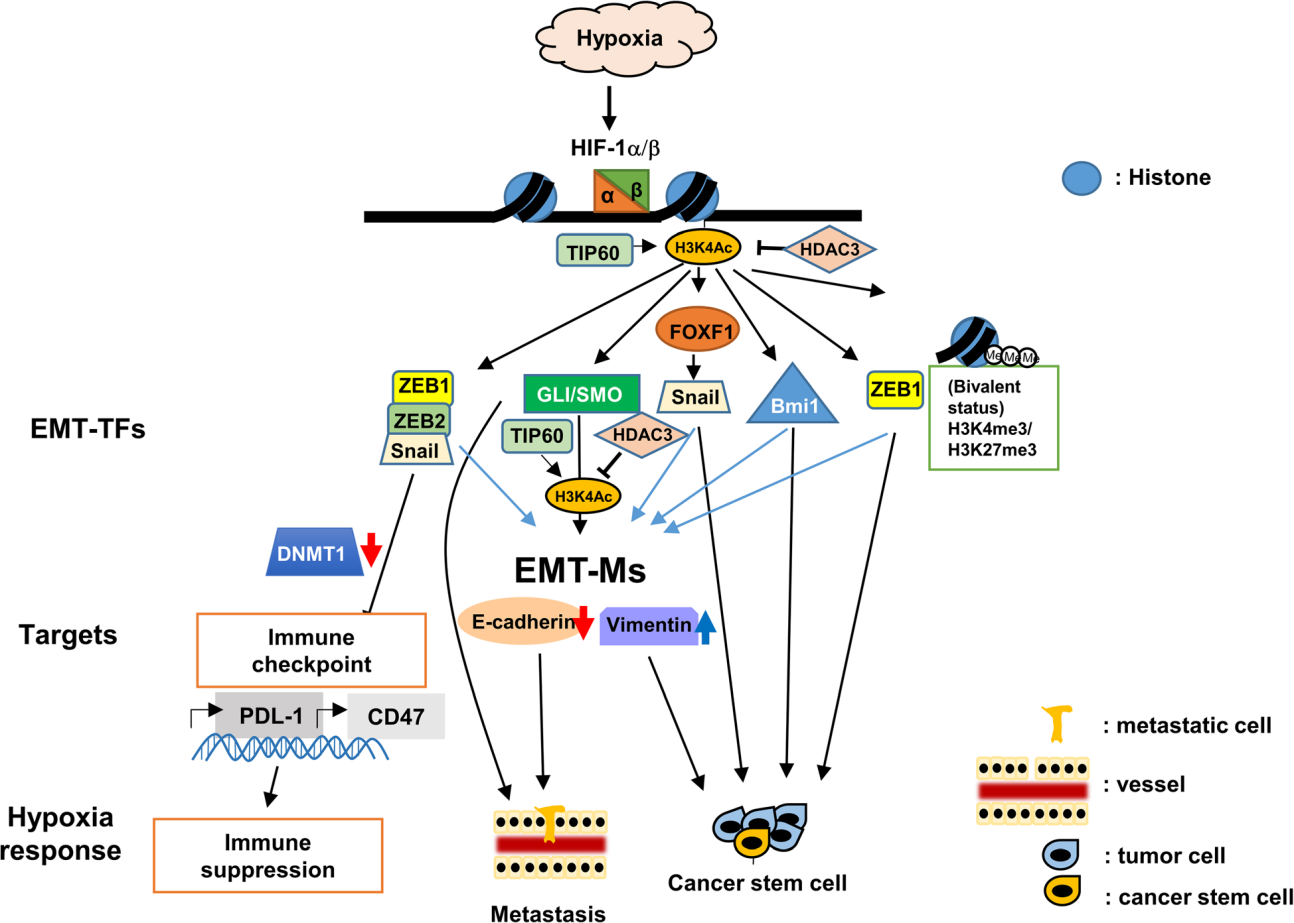
A summary of hypoxia-induced phenotypes through regulation of EMT transcription factors (EMT-TFs) and EMT markers (EMT-Ms). Three different phenotypes, including metastasis, cancer stemness, and immune suppression, are described. They are regulated by different signaling pathways depicted. Blue arrows indicate regulation of EMT marker expression (i.e. repression of epithelial genes and activation of mesenchymal genes)

**Fig. 2:**
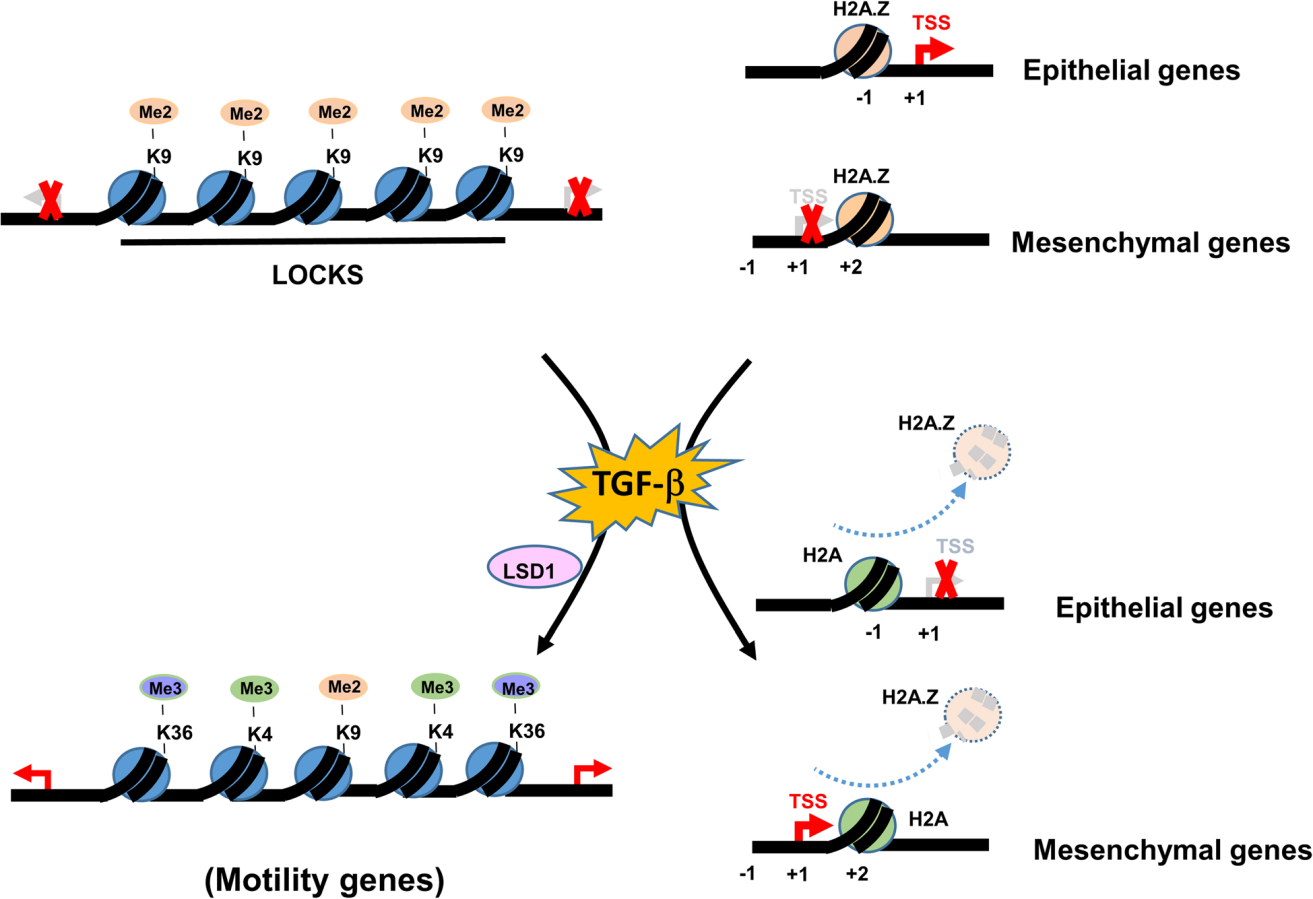
The chromatin changes induced by TGF-β during the EMT process. Overall changes in different histone marks (e.g. decrease in H3K9me2 and increase in H3K4me3/HeK36me3) and removal of a nucleosome variant (H2A.Z) in different positions are shown

**Fig. 3:**
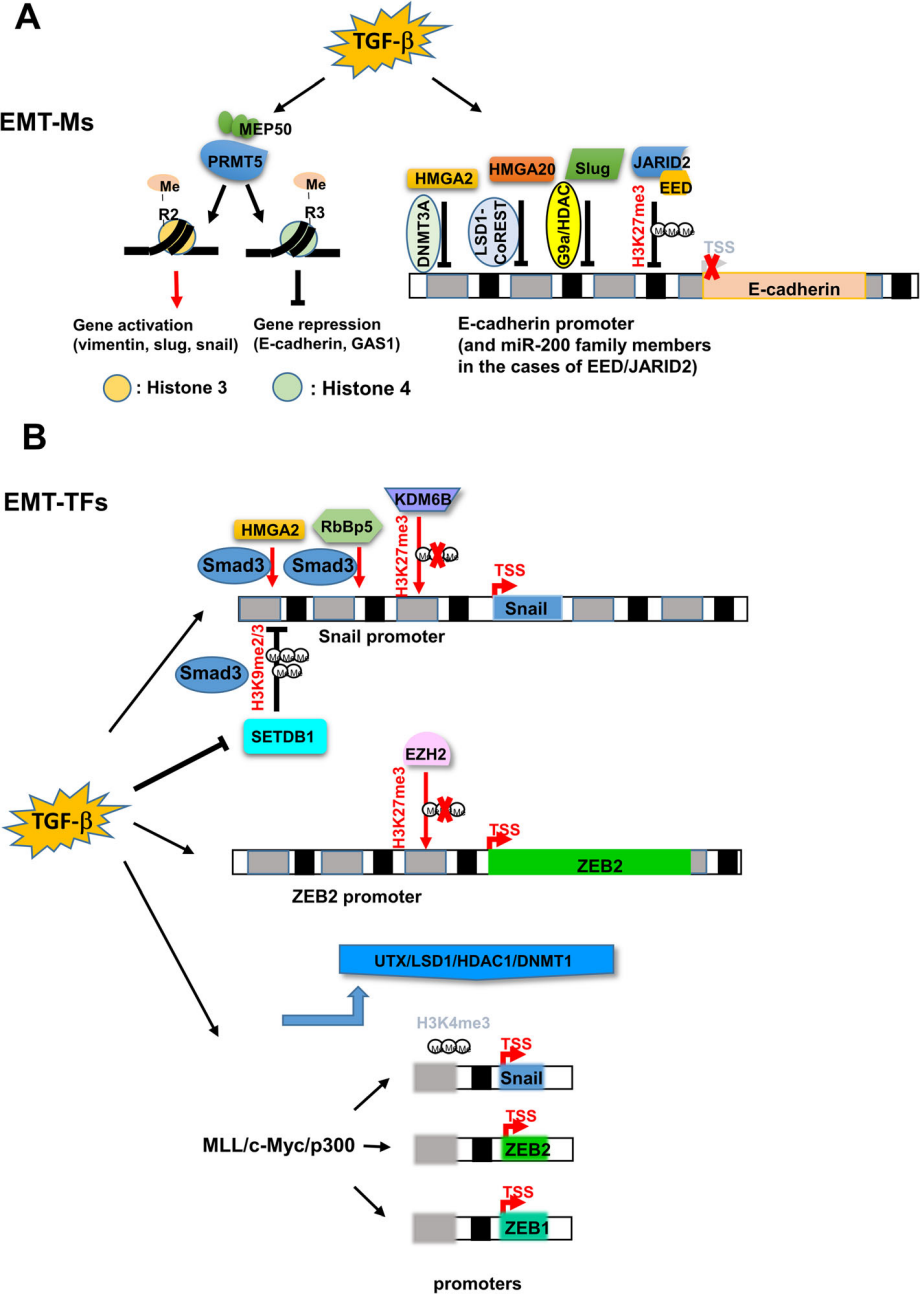
Different epigenetic signaling pathways regulated during TGF-β-induced EMT. **a** The regulation of EMT-Ms (mainly E-cadherin promoter) is shown through different epigenetic regulators. **b** The regulation of EMT-TFs (mainly Snail and ZEB2 promoters) by TGF-β is shown through different epigenetic regulators. Please notice that only the Smad3-SETDB1 pathway is shown in the repression of Snail promoter, in which SETDB1 expression is repressed by TGF-β. The opposite pathway of Smad3-CBP/p300 association triggered by TGF-β to activate Snail promoter is described in the text but not included in this panel

Since epithelial-mesenchymal transition is an important initial mechanism to mediate tumor metastasis, delineation of its molecular mechanisms will be key to handling tumor metastasis and treatment resistance derived from EMT. Epigenetic mechanisms are able to provide molecular understandings of EMT as well as suggest feasible therapeutic options. In this review, we focus on two major signaling events that trigger EMT: hypoxia and TGF-β. For hypoxia-induced EMT, we focus on the histone mark, H3K4Ac, that plays a pivotal role in the regulation of EMT markers and EMT transcription factors. H3K4Ac is a unique marker that allows genes regulated to be under bivalent status so their regulation of expression can maintain in a “poised transcription” state. This “poised transcription” state will permit gene expression to move flexibly between activation and repression. A summary of this aspect is depicted (Fig. 1). For TGF-β-induced EMT, we focus both on the global chromatin configuration (Fig. 2) and various chromatin modifiers (Fig. 3) that play a role in regulating gene expression. Both model systems will provide good examples for further exploration of new epigenetic mechanisms for other signaling pathways. Since new genome technologies (ATAC-seq, ChIP-seq, single-cell RNA-seq, etc) are being adopted to investigate different biological processes, we believe that new discoveries will be generated through using all these new genome technologies. New conceptual breakthrough may be able to provide new thinking in terms of the management of tumors with EMT involvement. As more findings have demonstrated the significance of epigenetic regulations of EMT, the relevant molecules may serve as not only biomarkers of tumor aggressiveness but also new potential targets for cancer therapy.

## Data Availability

Since the paper is a review article, there is no supporting experimental data.

## Abbreviations

ATAC-seq: Assay for transposase accessible chromatin-sequencing
ChIP: Chromatin immunoprecipitation
EMT: Epithelial-mesenchymal transition
EMT-Ms: Epithelial-mesenchymal transition markers
EMT-TFs: Epithelial-mesenchymal transition transcription factors
H3K27me3: Histone 3 lysine 27 trimethylation
H3K4Ac: Histone 3 lysine 4 acetylation
H3K9me3: Histone 3 lysine trimethylation
HIF-1α: Hypoxia-inducible factor 1-alpha
LSD1: Histone lysine demethylase 1
PRCs: Polycomb repressor complexes
